# Super Early Scan of PSMA PET/CT in Evaluating Primary and Metastatic Lesions of Prostate Cancer

**DOI:** 10.3390/molecules27144661

**Published:** 2022-07-21

**Authors:** Juanli Mao, Mingjun Gao, Bin Cui, Yingying Zhang, Xiaojiao Wang, Siyu Liang, Changjing Zuo, Peng Chen, Aisheng Dong

**Affiliations:** 1 Department of Nuclear Medicine, Shanghai Changhai Hospital, Shanghai 200433, China; juanlimaosh@163.com (J.M.); mingjungao2017@163.com (M.G.); cuibinwork@163.com (B.C.); zyymail126@126.com (Y.Z.); wxjresearch@yeah.net (X.W.); siyuliangsh@yeah.net (S.L.); 2 Department of Nuclear Medicine, Shanghai Tenth People’s Hospital, Tongji University School of Medicine, Shanghai 200072, China

**Keywords:** super early, ^68^Ga-PSMA-11 PET/CT, prostate cancer, primary lesions, metastasis

## Abstract

^68^Ga-prostate specific membrane antigen (PSMA)-11 PET/CT has been widely used in the diagnosis of prostate cancer (PCa); however, the urine lead shielding resulting from the urinary metabolism of tracers may obstruct the detection of surrounding metastasis. In this research, the additive value of super early scanning in diagnosing primary lesions and metastasis in the pelvic cavity was evaluated. Firstly, the differentiation efficiency of ^68^Ga-PSMA-11 PET scanned at 3 min post-injection (min P.I.) was measured in PSMA-positive (22rv1 cells) and PSMA-negative (PC3 cells) model mice. Secondly, 106 patients were scanned at 3 min P.I. for the pelvic cavity and then scanned as a standard protocol at 45 min P.I. In the results, the differential diagnosis of PSMA expression was completely reflected as early as 3 min P.I. for mice models. For patients, when correlated with the Gleason score, the quantitative results of the super early scan displayed a comparable correlation coefficient with the routine scan. The target to bladder ratios increased from 1.44 ± 2.40 at 45 min to 10.10 ± 19.10 at 3 min (*p* < 0.001) for the primary lesions, and it increased from 0.99 ± 1.88 to 9.27 ± 23.03 for metastasis. Meanwhile, the target to background ratios increased from 2.21 ± 2.44 at 3 min to 19.13 ± 23.93 at 45 min (*p* < 0.001) for the primary lesions, and it increased from 1.68 ± 2.71 to 12.04 ± 18.73 (*p* < 0.001) for metastasis. In conclusion, super early scanning of ^68^Ga-PSMA-11 PET/CT added referable information for metastasis detection in order to avoid disturbing tracer activity in the urinary system.

## 1. Introduction

Prostate cancer (PCa) is an epithelial malignancy of the prostate gland. Pathological types of prostate cancer include adenocarcinoma (acinar adenocarcinoma), ductal adenocarcinoma, urothelial carcinoma, squamous cell carcinoma and adenosquamous carcinoma, of which more than 95% are prostate adenocarcinoma. Some research has shown that prostate-specific membrane antigen (PSMA) is the characteristic membrane protein of PCa and is highly expressed in prostate cancer cells but rarely expressed in other tumors or normal tissue [1,2]. In addition, PSMA expression in PCa was positively correlated with the malignancy degree, which is normally quantified as the Gleason score (GS) and prostate antigen (PSA) [3,4]. Therefore, the corresponding PSMA-specific nuclear medicine diagnosis, such as ^68^Ga-PSMA-11 PET, ^18^F-PSMA-1007 PET and ^99m^Tc-HYNIC-PSMA SPECT, has been proposed as an important means of localization, staging and prognosis assessment; meanwhile, PSMA-specific radioligand therapy, such as ^177^Lu-PSMA-617 and ^225^Ac-PSMA-617, has provided an alternative for personalized therapy in metastatic castration-resistant prostate cancer [5,6,7].

As a commonly used PSMA-specific PET imaging agent, ^68^Ga-PSMA-11 has been rapidly promoted in clinical applications in densely inhabited districts, such as China and Europe, due to the convenience of in-house preparation and the availability of nuclides [8,9]. ^68^Ga-PSMA-11 PET/CT is a routine imaging diagnosis tool to delineate suspicious lesions, especially in subjects with tumor-negative biopsy, and to manage subjects in the follow-up period, even at low serum PSA values in biochemically recurrent PCa patients [10]. For the complementary diagnosis, ^68^Ga-PSMA-11 PET and MRI radiomics, such as the apparent diffusion coefficient (ADC) map, further increase the diagnostic performance in primary PCa patients [11].

Besides the clinical advantages regarding specificity, the popularized applications of PSMA PET/CT are limited by some objective features of images, such as the heavy uptake of glands and the signal interference from the bladder. Thus far, the avoidance of the interference from the bladder has been the main concern in practice, because of the adjacent connection of targeting tissues and metabolic organs. Some efforts have been made to solve this problem—for instance, adjustment of solubility in water and fat was found to be an effective method. When comparing ^18^F-DCFPyL with ^18^F-PSMA-1007, the increased fat solubility of PSMA-1007 contributes to the metabolism via hepatoenteral circulations [12,13]. However, the uncertainty of the metabolism has emerged, and the prolonged metabolism period affects the diagnosis efficiency for metastasis in the liver and intestinal tract to some extent. Another attempt aimed to speed up the excretion of tracers via the use of diuretics or by drinking more water, and these operations were clinically manifested by the clearance of the unwanted activity in the urinary tract, which in turn improved the contrast to noise ratios [14]. In fact, these interventions in urination will not lead to determinant changes to PSMA PET/CT diagnosis, but have an unknown influence on the tracer metabolism and corresponding accuracy of PSMA PET, because of the introduction of diuretics as confounding factors of the metabolic rate. There was also an attempt to use artificial intelligence to automatically detect pelvic lymph node metastases, so as to achieve high inter-observer sensitivity [15]; however, due to the fine structural connection between the bladder and surrounding tissue, as well as the partial volume effect of nuclear imaging, practical achievements could not be obtained.

Utilizing the time difference in the optimal scanning time points of distinct organs is a classic nuclear medicine solution, normally performed as a dual-phase scan or delayed scan, so as to meet the requirement in differentiating confused tissues [16,17]. In this research, a comparison among the commonly used PSMA PET precursors on the lipo-hydro partition coefficients was performed to evaluate the influence of labeling ligands; the feasibility of super early scanning was firstly evaluated in mice models, and then the super early scan was evaluated by adding an extra scan at 3 min post-injection (min P.I.) of ^68^Ga-PSMA-11 to the routine PSMA PET/CT.

## 2. Materials and Methods

### 2.1. Patient Selection

This research was approved by the Shanghai Changhai Hospital Ethics Committee (CHEC2019-090). From July 2020 to June 2021, 106 PCa patients were referred to the Department of Nuclear Medicine of Changhai Hospital for ^68^Ga-PSMA-11 PET/CT for clinical definitive diagnosis or staging, due to elevated PSA or Gleason score ≥ 5, or for evaluation of therapy response and restaging. For every patient, Gleason score and tPSA measured within 15 days were recorded. Detailed patient characteristics are shown in Table 1.

### 2.2. Preparation of PCa Models

For mice models, PSMA-positive (PSMA+) 22rv1 cells and PSMA-negative (PSMA-) PC3 cells were utilized to prepare the xenograft tumor models. NOD-SCID mice (*n* = 3 for each group, male, 8 weeks old) were used to establish subcutaneous xenograft tumor models. Firstly, the cells in the logarithmic growth stage were selected and re-suspended in 0.01 M PBS after digestion. The tumor cell suspension was mixed with Matrigel and temporarily stored in ice-cold water. The needle was inserted into the armpit at an oblique angle of 45 degrees, and held in the subcutaneous position to inject 100 µL tumor cell (1 × 10^6^ cells for each mouse) mixture into the lateral subcutaneous area of the right upper limb. The needle was further kept for a while until the matrix glue solidified. There was a period of 10 days for PC3 xenografts to reach 200–500 mm^3^, and 45–60 days for 22rv1 xenografts to reach 200–500 mm^3^.

### 2.3. Measurement of Lipo-Hydro Partition Coefficient

First, 100 μg PSMA PET precursor, including PSMA-11, PSMA-617 and PSMA-1007, was dissolved in a mixture of 500 μL ultrapure water and 500 μL octanol, and then vortexed and oscillated for 5 min at 25 °C. The mixture was left to stand and layered for a period of time, and 100 μL solutions were acquired from the superstratum and substratum separately to measure the absorbance at 220 nm. The lipo-hydro partition coefficient was recorded as Log P and calculated as Log P = Log (Co/Cw), where Co stands for the concentration in the organic phase, and Cw stands for the concentration in water.

### 2.4. ^68^Ga-PSMA-11 PET/CT

^68^Ga-PSMA-11 was prepared in house in a hot cell for synthesis. The specific activity of the routine production of ^68^Ga-PSMA-11 was around 11.1 MBq/µg with radiochemical purity of more than 99%.

For mice models, mice were injected with 25 µL 3% pentobarbital sodium as an anesthetic. Mice were injected with 3.7 MBq ^68^Ga-PSMA-11 and scanned at 3 min and 45 min P.I. using the clinically used PET/CT scanner (Biograph64, Siemens, Germany, the same below). PET/CT scans started with a low-dose CT scan followed by a PET scan with parameters as below: for CT, tube voltage: 120 kV; tube current: 35 mA; pitch: 1.0; reconstructed layer thickness: 1 mm; for PET, the whole-body images were acquired for one bed within one minute.

For patients, after resting for 15 min, the patient laid flat on his back on the scanning table and ^68^Ga-PSMA-11 was injected as 2.00–2.5 MBq/kg body weight intravenously. The positioning scan and the setting of the PET scan were completed within 3 min after injection, and the single-bed PET scan was performed during 3 to 5 min P.I. (2 min in all). The scanning range was limited to the pelvic cavity. After the completion of super early scanning, the patient rested for 40 min, drank water, urinated and then underwent the routine PET/CT examination at 45 min P.I. In addition, the delayed scan between 90 and 120 min was performed if necessary. During the scanning, the patient’s arms were placed at the sides of the body, and the scan ranged from the head to the middle femur; a CT scan of the body (voltage: 120 kV, reconstruction layer thickness: 3 mm) was firstly carried out, and then a PET scan of the body (6–7 beds in total, 3 min for each bed) was carried out. The current intensity of the tube was automatically adjusted according to the thickness and density of the subject.

After data attenuation correction, iterative reconstruction was carried out, and the image automatic alignment fusion display was carried out using the MedEx workstation system (Beijing MedEx Technology Co., Ltd., Beijing, China), so as to obtain the horizontal plane, coronal plane and sagittal plane images and maximum intensity projection (MIP) images.

### 2.5. Image Interpretation of ^68^Ga-PSMA-11 PET/CT

^68^Ga-PSMA-11 PET/CT images were double-blind read by two experienced physicians. For normal ^68^Ga-PSMA-11 PET/CT imaging, normal physiological uptake can be seen in the prostate, salivary gland, lacrimal gland, submandibular gland, liver, small intestines and pancreas; irregular uptake can be seen in bone and joint degeneration; physiological concentration can be seen in the gallbladder, kidney, ureter and bladder.

The super early scan at 3 min P.I. was mainly evaluated with respect to the adjacent organs, bones and lymph nodes in the pelvic cavity, supplying more valid and valuable information. Routine and delayed PET/CT images remained as the main basis for the diagnosis, comprehensively for the primary lesion, distant lymph node involvement and metastases. When the uptake of ^68^Ga-PSMA-11 in both lobes of the prostate significantly increased, it was diagnosed as prostate cancer involving both lobes of the prostate. Prostate cancer was diagnosed as breaking through the capsule when the increased PSMA uptake caused local swelling of the prostate capsule, the outer edge of the prostate lost its smooth shape, and there was local irregularity, continuity interruption and roughness. Similarly, local abnormal uptake foci on adjacent organs or bones other than physiological uptake or concentrated foci were diagnosed as metastasis or tumor invasion. Lymph node metastasis was diagnosed when the length and diameter of regional lymph nodes exceeded 10 mm or the PSMA uptake of regional lymph nodes was significantly higher than the background.

For pelvic metastases, the three closest metastases to the primary lesion or prostatectomy area were recorded, and those subjects with more than three metastases were recorded as three. Correlation analysis was conducted based on the closest metastasis to the prostate.

### 2.6. Statistical Analysis

The quantification of tracer uptake was correlated to GS, and data were divided into super early scan and routine scan, and into primary and metastasis. For the primary and metastatic lesions, SUV_max_3_ was correlated to SUV_max_45_. Accordingly, the correlations of paired samples were described with the correlation coefficient (r) and significance (p).

Target to background (T/BA) and target to bladder (T/BL) ratios were calculated to evaluate the differential efficiency. For SUV_max_ or T/B ratios that were acquired at 3 min P.I. and 45 min P.I., the paired t-test was used to compare the values of primary lesions, metastatic lesions, bladder, primary to bladder and metastasis to bladder. Statistics were analyzed with SPSS 26.0 (IBM, New York, NY, USA), and a *p*-value less than 0.05 was considered significant.

## 3. Results

### 3.1. Lipo-Hydro Partition Coefficient

The commonly used PSMA PET precursors, such as PSMA-11, PSMA-617 and PSMA-1007, were of distinct lipo-hydro partition coefficients, which determined the metabolic pathway as a crucial factor. As proven in this study, PSMA-11 was of a moderate Log P as 5.125 and utilized as the representative precursor in evaluating the super early scan of PSMA PET. Additionally, the values of Log P of PSMA-617 and PSMA-1007 were 1.758 and 8.375.

### 3.2. Verification of Diagnostic Efficiency of PSMA PET in Model Mice

**Figure 1:**
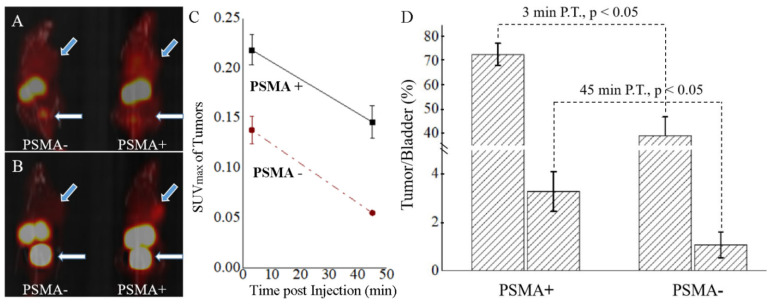
MIP images of PSMA-negative and PSMA-positive xenograft models that were acquired at 3 min (**A**) and 45 min (**B**) P.I., where blue arrows point to the xenografts, and white arrows point to the bladder. (**C**) The absolute values of tracer uptake at 3 and 45 min P.I.; the corresponding percentages for tumor to bladder ratios at 3 min P.I. and 45 min P.I. are summarized in columns (**D**).

Figure 1, acquired with mice models, fully exhibits the effective avoidance of shielding from the bladder. For the MIP images acquired with the super early scan at 3 min P.I., only the tumor, kidney and bladder were the differentiable tissues, while the bladder was fully filled with radiotracer-containing urine at 45 min P.I. (Figure 1A,B). The differential diagnosis of PSMA+ and PSMA- was exhibited as early as 3 min P.I., but the SUV_max_ consisted of the specific tracer uptake and blood fluids, where the latter decayed in the routine scan at 45 min P.I. (Figure 1C). The T/BL ratios were 72.75 ± 4.60% for PSMA+ models and 39.11 ± 7.73% for PSMA- models, where a comparable level of tracer uptake in the bladder in distinct models was recorded (Figure 1D). The significant difference between PSMA+ and PSMA- models meant that the tracer uptake resulted from not only the rich blood supply but also the variation in PSMA expression. For the MIP images acquired at 45 min P.I., the non-specific uptake of the PSMA- xenograft decreased to a level similar to the background, and the specific uptake of the PSMA+ xenograft was maintained at a differentiable level, but most of the radioactivity was focused in the bladder. The T/BL ratios dramatically decreased to 3.29 ± 0.82% for PSMA+ models and 1.08 ± 0.53% for PSMA- models. In these situations, with low T/BL ratios, the partial volume effect of the bladder will lead to a shielding effect on the adjacent lesions, whether a PSMA+ one or a PSMA- one.

### 3.3. Clinical Findings of Super Early ^68^Ga-PSMA-11 PET

**Figure 2:**
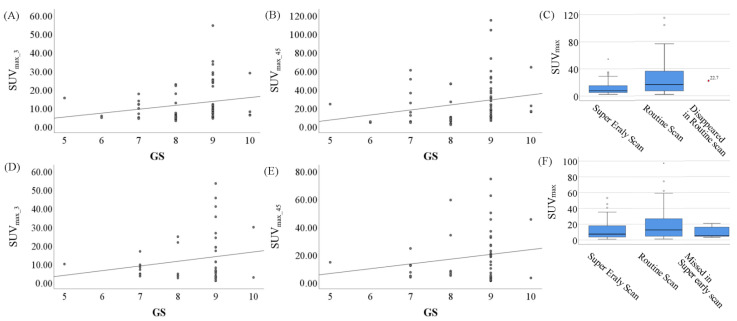
Correlation between Gleason scores and SUV_max_, including the primary lesion at 3 min (**A**) and 45 min (**B**) P.I., and the corresponding box–whisker plots (**C**) of SUV_max_ acquired at super early scan and routine scan, as well as the disappeared lesion in routine scan. The same results of the metastatic lesions are exhibited in (**D**–**F**) (the missed lesion in super early scan).

There was a comparable correlation between GS and SUV_max_ that were acquired at 3 min and 45 min P.I. For the primary lesions (Figure 2A,B), the correlation coefficients were 0.217 for SUV_max_3_ and 0.237 for SUV_max_45_. There were significant increases in SUV_max_ from 3 min P.I. to 45 min P.I. For the primary lesions (Figure 2C), there was a significant difference between SUV_max_3_ and SUV_max_45_ (11.75 ± 9.57 vs. 24.78 ± 23.26, *p* < 0.001), and SUV_max_3_ was significantly correlated with SUV_max_45_ (r = 0.863, *p* < 0.001). For the super early scan of 81 PCa subjects without radical prostatectomy (RP), 78 subjects presented detectable in situ lesions, while three subjects showed PSMA- in situ lesions. For the routine scan, 77 subjects presented detectable in situ lesions. For the subject with a radioactive signal that faded away during the 40 min rest period (Figure 2C), a lesion of SUV_max_ = 22.7 was detected in the super early scan.

For the metastatic lesions (Figure 2D,E), the correlation coefficients between GS and SUV_max_ were 0.192 for SUV_max_3_ and 0.187 for SUV_max_45_. For the metastatic lesions (Figure 2F), there was a significant difference between SUV_max_3_ and SUV_max_45_ (12.58 ± 12.14 vs. 20.55 ± 20.12, *p* < 0.001), and SUV_max_3_ was significantly correlated with SUV_max_45_ (r = 0.872, *p* < 0.001). For the super early scan of PCa subjects with GS, 56 subjects presented one or more detectable metastatic lesions, and a total of 76 metastases were detected, while 25 subjects presented no PSMA+ findings on metastases. For the routine scan, 61 subjects presented detectable metastatic lesions, and a total of 96 metastases were detected, while only 20 subjects presented no PSMA+ findings on metastases. For the five subjects that presented missed metastatic lesions, the detected lesions in the routine scan were of a significantly lower SUV_max_ than the complete cohort (Figure 2F and Table 2). The low level of PSMA expression, small size and poor blood supply of some lesions may have contributed to the missing metastatic lesions in the super early scan. Especially for the pelvic lymph nodes, the high ^68^Ga-PSMA-11 uptake at 45 min P.I. was more supportive of the diagnosis of metastasis.

In addition, in the bladder, there was a significant difference between SUV_max_3_ and SUV_max_45_ (9.72 ± 21.29 vs. 37.62 ± 37.52, *p* < 0.001), and SUV_max_3_ was significantly correlated with SUV_max_45_ (r = 0.232, *p* = 0.021).

**Figure 3:**
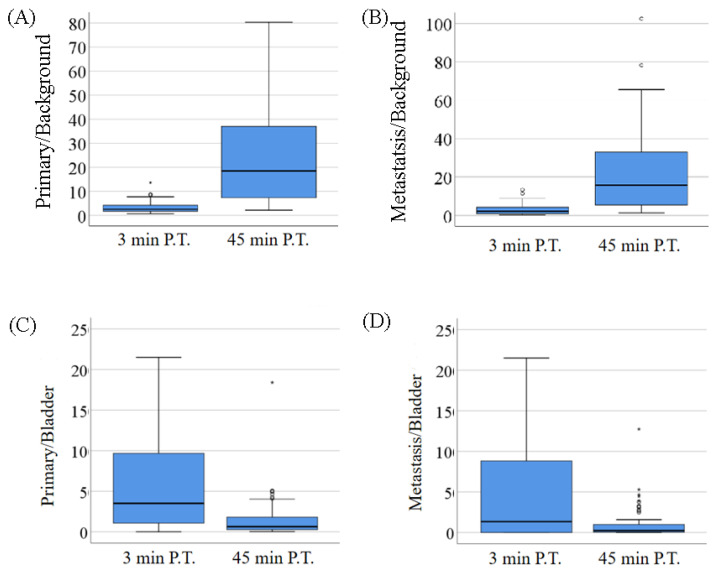
Box–whisker plots of the main findings, including tumor to background ratios of the primary lesions (**A**), metastases (**B**), and tumor to bladder ratios of the primary (**C**) and metastases (**D**). The T/BL values higher than 25 are not displayed in figures.

Figure 3 provides a series of box–whisker plots of the main findings on T/background and corresponding T/bladder ratios. There were significant increases in target to background (iliac artery) ratios from 3 min to 45 min P.I., meaning a higher contrast ratio and higher efficiency in differentiating lesions with background in the pelvic cavity at 45 min P.I. For the ratios of primary lesion to background (Figure 3A), there was a significant difference between P/BA_3 and P/BA_45 (2.21 ± 2.44 vs. 19.13 ± 23.93, *p* < 0.001), and P/BA_3 was significantly correlated with P/BA_45 (r = 0.751, *p* < 0.001). For the P/BA_3 ratios acquired from the super early scan, the range was almost as low as 0 and as high as 13.60. For the ratios of metastatic lesions to bladder (Figure 3B), there was a significant difference between M/BA_3 and M/BA_45 (1.68 ± 2.71 vs. 12.04 ± 18.73, *p* < 0.001), and M/BA_3 was significantly correlated with M/BA_45 (r = 0.678, *p* < 0.001). For the P/BA_3 ratios acquired from the super early scan, the range was almost as low as 0 and as high as 13.33.

There were significant decreases in target to bladder ratios from 3 min to 45 min P.I., meaning a higher contrast ratio and higher differential efficiency in diagnosing lesions in the pelvic cavity at 3 min P.I. For the ratios of primary lesions to bladder (Figure 3C), there was a significant difference between P/BL_3 and P/BL_45 (10.10 ± 19.10 vs. 1.45 ± 2.40, *p* < 0.001), and P/BL_3 was significantly correlated with P/BL_45 (r = −0.719, *p* < 0.001). For the P/BL_3 ratios acquired from the super early scan, the range was almost as low as 0 and as high as 84.33 (the values higher than 25 are not displayed in Figure 3C). For the ratios of metastatic lesions to bladder (Figure 3D), there was a significant difference between M/BL_3 and M/BL_45 (9.27 ± 23.03 vs. 0.99 ± 1.88, *p* = 0.002), and M/BL_3 was significantly correlated with M/BL_45 (r = −0.728, *p* < 0.001). For the P/BL_3 ratios acquired from the super early scan, the range was almost as low as 0 and as high as 151.67 (the values higher than 25 are not displayed in Figure 3D). It is notable that there were seven subjects with no urine burden (fully blank in nuclear imaging) at 3 min P.T., and they were excluded from the statistics of T/BL ratios.

**Figure 4:**
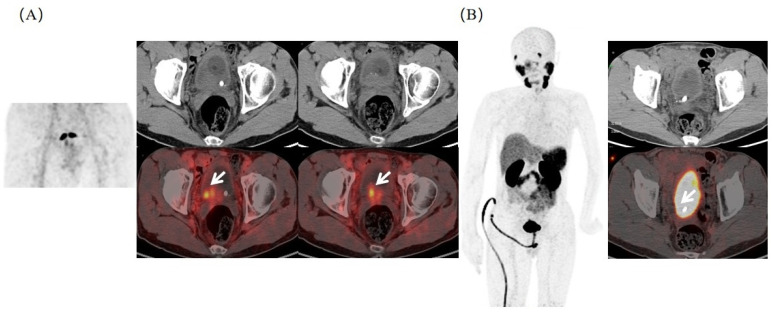
^68^Ga-PSMA-11 PET/CT in a 65-year-old PCa patient after endocrine therapy with elevated tPSA (42.18 ng/mL). (**A**) Focal increased ^68^Ga-PSMA-11 uptake (SUV_max_ = 22.7, T/BL ratio = 15.1) on the posterior wall of the bladder on fused axial PET/CT at 3 min P.I. (white arrow). (**B**) Corresponding bladder images at 45 min P.I., with markedly increased tracer accumulation. Differentiation of lesions and physiologic urethra activity was not possible.

For most subjects, the SUV_max_ increased from the super early scan to routine scan. In fact, there were seven subjects with no urine burden in the super early scan, but three in situ lesions and four metastatic lesions were detected. More importantly, super early imaging was more valuable in diagnosing prostate cancer when invading surrounding organs. The bladder was not filled with tracers at 3 min P.I., which can demonstrate the bladder structure and the relationship between surrounding tissues. Therefore, when prostate cancer invaded the posterior wall of the urinary bladder, higher focal ^68^Ga-PSMA-11 uptake could be observed on PET/CT images at 3 min P.I., but this was indistinguishable at 45 min P.I. (Figure 4). The hidden prostate cancer invasion of the urinary bladder in the routine scan could be detected in the super early scan, which may aid in modifying further treatment protocol management.

**Figure 5:**
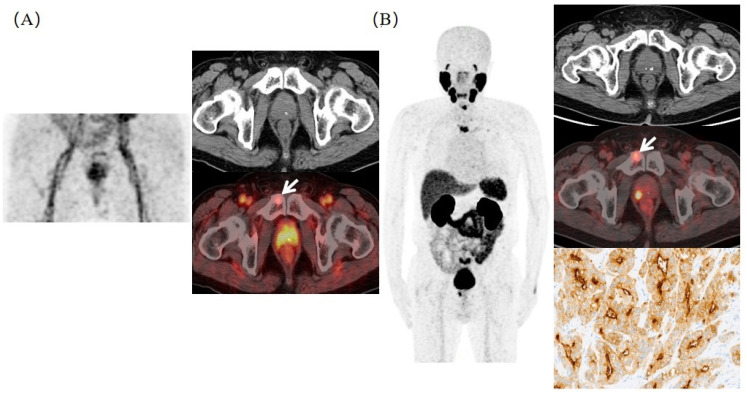
Typical ^68^Ga-PSMA-11 PET/CT images in detecting osseous metastasis. (**A**) The right pubic bone metastasis (white arrow) was visible at 3 min P.I. (SUV_max_ = 5.1). (**B**) Corresponding bone metastasis (white arrow) also displayed at 45 min P.I. (SUV_max_ = 8.2), which was confirmed in pathological results.

Besides lymphatic metastasis, pelvic osseous metastases were also commonly detected in patients with advanced prostate cancer. A super early PSMA PET scan can also provide important information on osseous metastases. As shown in Figure 5, multiple bone metastases were observed in a 78-year-old PCa patient after endocrine therapy (tPSA = 14.28 ng/mL) in ^68^Ga-PSMA-11 PET/CT. The super early PSMA PET scan suggested the right pubic bone metastasis, which was consistent with the images at 45 min P.I.

## 4. Discussion

In this research, although mice did not present urine during the 45 min observation period, the avoidance of bladder shielding was fully exhibited by the increase in the T/BL ratios of the PSMA+ models. There was a 22-fold difference between the 3 min and 45 min data. In addition, the high uptake of the kidneys further proved the existence of strong shielding from the dynamic urinary metabolism. In clinical findings, PSMA specificity of tracer uptake at the super early phase was verified with the correlation between tracer uptake and GS, as well as the correlation between tracer uptake and the corresponding values of 45 min. The specificity confirmed the validity of early PSMA PET.

Resulting from the difference in in vivo circulation, there was a time difference in the start of urine burden and PSMA-specific uptake [18]. It has been demonstrated that early dynamic imaging in the first 5 min P.I. enables the tumor to be distinguished from bladder activity in ^68^Ga-PSMA-11 PET/CT, but the performance of dynamic imaging is quite demanding compared to a routine static scan. Moreover, in the clinical results of the diagnosis of primary lesions, there was comparable efficiency, but the routine scan was of a higher detection rate in diagnosing metastases. Although the absolute value of SUV_max_ in the super early scan was low, the obvious added value was exhibited by the enhanced T/BL ratios, increasing the confidence in the lesion diagnosis. For instance, due to the connected structure of the prostate and bladder, the increased T/BL was meaningful in diagnosing primary lesions.

Performing diagnosis on the basis of distinct phases is commonly used in nuclear medicine imaging, such as the dual-phase ^99m^Tc-MIBI SPECT/CT scan in diagnosing thyroid disease and the delayed ^18^F-FDG PET/CT scan in differentiating tumors and inflammation [19,20]. Currently, the signal interference from the urinary tract and liver is still the main focus and difficulty in nuclear medicine diagnosis [21,22]. The imaging and evaluation of prostate cancer and its local metastases could be obstructed by the high radioactivity inside the urinary bladder. Detection of recurrent disease in cases of invasive bladder cancer can be significantly improved by using FDG PET/CT with a delayed scan following forced diuresis and oral hydration. However, methods such as the administration of diuretics have only shown limited success [23]. As has been reported, adding an early scan (5 min P.I.) to the routine examination can result in a dual-phase scanning mode in the ^68^Ga-PSMA-11 PET/CT of prostate cancer, which is a promising approach to reduce the interference of radiotracer excretion in diagnosis [24]. Interestingly, compared with 5 min P.I., there was sufficiently high lesion uptake as early as 3 min P.I., and super early imaging obtained before radiotracer accumulation within the urinary bladder seemed more helpful for the evaluation of lesions on or close to the bladder.

When compared with the other methods of improving the diagnosis of metastasis in the pelvic cavity, the addition of the super early scan caused no interference with the injection, dosage and metabolism of ^68^Ga-PSMA-11, retaining the advantages of ^68^Ga-PSMA-11 PET/CT. In contrast, diuretics and modification of the precursor can add uncertainty to PCa diagnosis, and more clinical trials are needed to clarify the details of operation and image interpretation. It is notable that radiation exposure was not doubled in our study because the super early scan was limited to the pelvic cavity, leading to a 16.7% or 14.3% increase in radiation exposure.

In addition, regarding the imaging of lesions with targets expressed on blood vessels or cell membranes, such as RGD-targeted and VEGF-targeted imaging, there is also potential to distinguish the lesions from the metabolic organs at the super early stage, benefiting from the early implementation of targeting [25,26].

## 5. Conclusions

Super early scanning of ^68^Ga-PSMA-11 PET/CT significantly increased the target to bladder ratios and increased the differential efficiency in diagnosing primary lesions, but it yielded a lower SUV_max_ as a consequence, and lower detective efficiency in diagnosing metastasis. With the comprehensive consideration of the additive radiation exposure and additive clinical value, a super early PSMA PET scan is recommended as an effective supplement to routine scans, especially for patients with clinical symptoms or other imaging data suggesting the invasion of adjacent organs (such as the posterior wall of the urinary bladder or the anterior wall of the rectum), and osseous and lymphatic metastasis in the pelvic cavity will benefit from the precise control of scanning time points. Super early scanning has the potential to avoid the interference caused by the metabolism of tracers through the urinary system and reduce missed diagnosis, which is more conducive to guiding clinical staging and treatment planning.

## Figures and Tables

**Table 1:** Patients’ characteristics.

Items	Groups	Number or Value
Patients	-	106
	Radical prostatectomy	25
Age (years)	-	67.1 (57–81) *
Gleason score	NAN	25
	5	1
	6	2
	7	13
	8	18
	9	43
	10	4
tPSA (ng/mL) **	-	2.47 (0.02–100) *

* range is provided in brackets; ** 40 patients were of no tPSA within 15 days.

**Table 2:** The characteristics of five subjects that presented missed metastatic lesions in 3 min P.I.

Patients	Gleason Score	Metastatic Lesions at 45 Min P.I.		
		Size (cm)	Location	SUV_max_45_
1	7	4.3 × 3.5	right pelvic wall	16.2
2	7	1.2 × 1.1	pelvic lymph nodes	3.6–5.2
3	8	1.9 × 1.5	pelvic lymph nodes	2.6–4.8
4	9	4.8 × 2.9	left rectus abdominis	3.5
5	9	6.1 × 5.6	second lumbar vertebra	5.3

## Data Availability

Not applicable.

## Sample Availability

Samples of the compounds, including PSMA-11, PSMA-617 and PSMA-1007, are available from the authors.
